# Cardiac resynchronisation therapy in optima forma

**DOI:** 10.1007/s12471-022-01683-x

**Published:** 2022-04-07

**Authors:** S. A. J. Timmer, T. Germans

**Affiliations:** Department of Cardiology, Northwest Clinics, Alkmaar, The Netherlands

## Answer

The electrocardiogram in Fig. 2 shows atrioventricular (AV) sequential pacing at a rate of 60 bpm with QRS duration ~90 ms. The QRS morphology is consistent with conduction system pacing, in particular selective His bundle pacing (HBP) with correction of LBBB.

HBP is a cardiac pacing technique with the potential of preserving or restoring normal His-Purkinje activation in the presence of AV conduction block or BBB. A prerequisite for successful HBP in the setting of LBBB is the presence of a proximal or intra-His conduction block, which can be circumvented by pacing the distal His bundle and thereby recruiting the otherwise ‘healthy’ left-sided conduction system. LBBB can be corrected with HBP in up to 80% of patients depending on the site of the block [1], and the conduction block site correlates reasonably well with surface ECG. The Strauss criteria have a positive predictive value of 71% for identifying patients eligible for corrective HBP [2]. Although, as of yet, there is no clear benefit of HBP over traditional biventricular pacing because this has not been investigated properly, HBP is potentially the ultimate form of cardiac resynchronisation therapy [3].

**Fig. 1:**
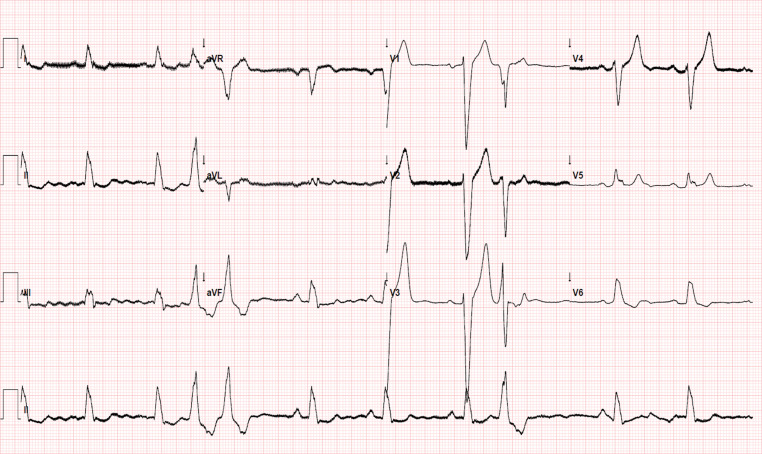
Preoperative electrocardiogram

**Fig. 2:**
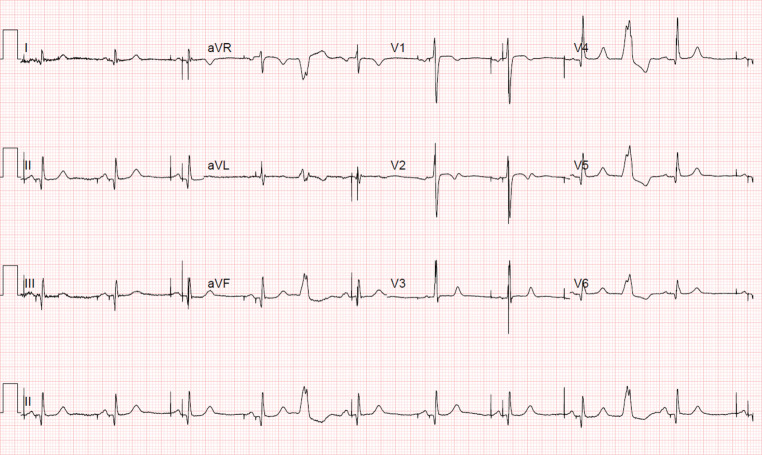
Postoperative electrocardiogram

**Fig. 3:**
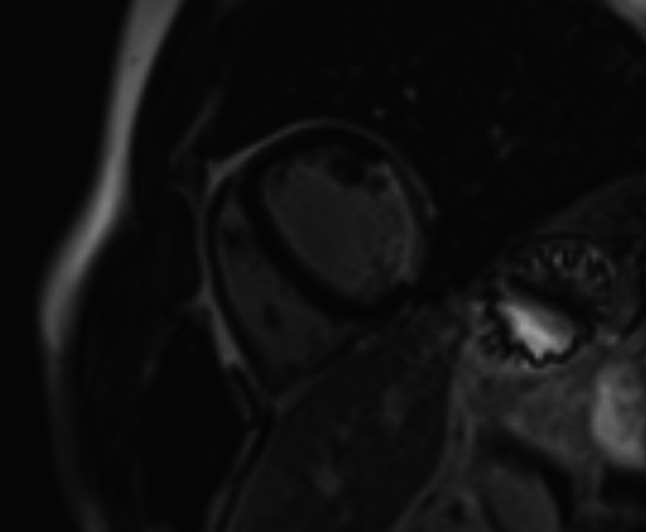
Cardiovascular MRI with gadolinium contrast showing transmural myocardial infarction of inferolateral wall

In our patient, pathological Q waves in the inferior and lateral leads, which were previously not visible (see Fig. 1), were revealed postoperatively in conjunction with a dominant R wave in the right precordial leads (Fig. 2), as a result of normalisation of ventricular electrical activation. These ECG findings could be related to the findings of cardiac magnetic resonance imaging (MRI), which revealed transmural inferolateral myocardial infarction (Fig. 3).

## References

[1] Moriña-Vázquez P, Moraleda-Salas MT, Manovel-Sánchez AJ (2020). Early improvement of left ventricular ejection fraction by cardiac resynchronization through His bundle pacing in patients with heart failure. Europace.

[2] Upadhyay GA, Cherian T, Shatz DY (2019). Intracardiac delineation of septal conduction in left bundle-branch block patterns. Circulation.

[3] Upadhyay GA, Vijayaraman P, Nayak HM (2019). On-treatment comparison between corrective his bundle pacing and biventricular pacing for cardiac resynchronization: a secondary analysis of the his-SYNC pilot trial. Heart Rhythm.

